# A global reference model of Curie-point depths based on EMAG2

**DOI:** 10.1038/srep45129

**Published:** 2017-03-21

**Authors:** Chun-Feng Li, Yu Lu, Jian Wang

**Affiliations:** 1 Institute of Marine Geology and Resources, Ocean College, Zhejiang University, Zhoushan 316021, China; 2 School of Ocean and Earth Sciences, Tongji University, Shanghai 200092, China; 3 Key Laboratory of Crustal Dynamics, Institute of Crustal Dynamics, China Earthquake Administration, Beijing 100085, China; 4 Laboratory for Marine Mineral Resources, Qingdao National Laboratory for Marine Science and Technology, Qingdao 266237, China

## Abstract

In this paper, we use a robust inversion algorithm, which we have tested in many regional studies, to obtain the first global model of Curie-point depth (GCDM) from magnetic anomaly inversion based on fractal magnetization. Statistically, the oceanic Curie depth mean is smaller than the continental one, but continental Curie depths are almost bimodal, showing shallow Curie points in some old cratons. Oceanic Curie depths show modifications by hydrothermal circulations in young oceanic lithosphere and thermal perturbations in old oceanic lithosphere. Oceanic Curie depths also show strong dependence on the spreading rate along active spreading centers. Curie depths and heat flow are correlated, following optimal theoretical curves of average thermal conductivities K = ~2.0 W(m°C)^−1^ for the ocean and K = ~2.5 W(m°C)^−1^ for the continent. The calculated heat flow from Curie depths and large-interval gridding of measured heat flow all indicate that the global heat flow average is about 70.0 mW/m^2^, leading to a global heat loss ranging from ~34.6 to 36.6 TW.

Our planet Earth is under constant cooling and differentiation since its origin. Its current thermal state and heat loss can be estimated from surface heat flow measurements^1^,^2^,^3^,^4^,^5^,^6^. However, heat flow estimates have evident drawbacks; they are often measured at sparsely and irregularly distributed sites, and they are strongly affected by shallow hydrothermal circulation and therefore are limited in inferring deep thermal structure of the lithosphere. These uncertainties put quite different estimates of global heat loss. In one early study^1^, and in some recent calibrations minimizing the hydrothermal effects^3^,^4^, the global heat loss was estimated at about 40–47 TW (or 4.4–4.7 × 10^13^ W). Other reappraisals of global heat flow database, however, concluded that global conductive heat loss falls in the range from 29 to 34 TW^2^,^5^,^6^. The differences between these estimates vary considerably.

An alternate and independent method of studying deep thermal structure and global heat loss is by detecting Curie depths from inversion of surface total field magnetic anomalies^7^,^8^,^9^,^10^,^11^,^12^,^13^,^14^. Global coverage of magnetic anomalies is being constantly improved in recent years^15^,^16^, and this allows it possible to map the global Curie isotherm in high-resolution, assuming that the Curie-point temperature is constant globally around 550 °C^17^,^18^ and that lateral compositional variations exert neglectable influence on the Curie temperature.

There are several different technical schemes of Curie depth inversion from magnetic anomalies, and even for the same method the selected inversion parameters can also vary among scientists^7^,^8^,^9^,^10^,^11^,^12^,^13^,^14^. Furthermore, previous studies focus only on a particular region of the Earth. These facts render it impossible the global comparison of Curie depth results.

In early regional studies, we have successfully automated the centroid method based on radially-averaged amplitude spectrum to obtain high-resolution Curie depths^9^,^10^,^11^. This saves considerably the computational time. Now we can naturally apply this algorithm to the Earth Magnetic Anomaly Grid of 2-arc-minute resolution (EMAG2, http://geomag.org/)^16^ to obtain the first global reference model of Curie-point depths (GCDM).

## Implementation of the Algorithm

The Earth’s surface from −75° to 75° latitudes is divided into 72 zones, and the two polar zones are treated separately. Within each zone, we transform the magnetic data from geographical coordinate to Cartesian coordinate, before gridding the data in a constant 2.6 km interval using the minimum curvature method, which iteratively solves a set of differential equations to minimize the total second horizontal derivative and honor input data^19^. We then estimate Curie depths using three different window sizes, 98.8 × 98.8 km^2^, 195.0 × 195.0 km^2^, and 296.4 × 296.4 km^2^. The size of 296.4 × 296.4 km^2^ is large enough to capture the deepest Curie depths around 50 km, and is larger than in most existing applications of the algorithm. The moving steps for these three window sizes are 49.4 km, 97.5 km, and 98.8 km, respectively. As did before^20^, we take the average of Curie depths from the three windows as the final Curie depth model. Taken to be constant at 3, the scaling factor of 3D fractal magnetic sources,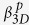 is defined by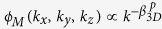, in which 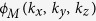 is the 3D power spectrum of the magnetization, *k*_*x*_, *k*_*y*_, and *k*_*z*_ are wavenumbers in x, y, and z directions, respectively, and their Euclidean norm


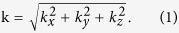


**Figure 1:**
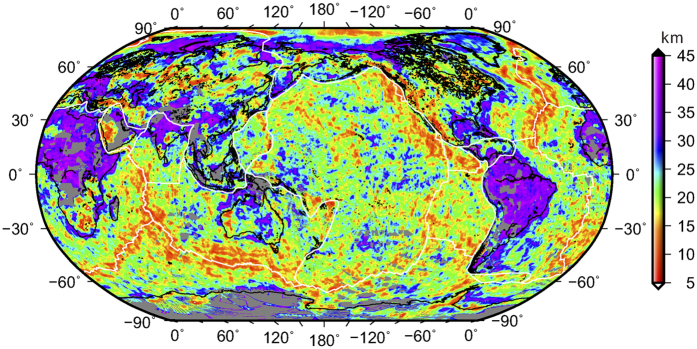
The global reference Curie-point depth model (GCDM) estimated in this study from the Earth Magnetic Anomaly Grid of αarc-minute resolution (EMAG2^16^). White lines mark the major plate boundaries^32^. Map is generated using the USGS potential field software^29^,^30^ and software GMT version 5.2.1 (http://gmt.soest.hawaii.edu/)^31^.

The datum altitude (4 km) of the EMAG2 is subtracted from the estimated Curie depths to be in reference to the geoid (Fig. 1).

We applied the standard fast Fourier transform scheme in estimating spectra, but with careful consideration and treatment of boundary effects and short-wavelength tails^21^. We use the wavenumber band of ~0.005–0.03 1/km for estimating the centroid depths, not just the first slope for stability reasons. It is true that same spectral wavenumber range is not applicable for every window. Our algorithm can judge automatically if the first (smallest wavenumber) point is smaller than the second in the spectra and, if so, does not account this point in the calculation, because theoretically this cannot happen^11^. In each window, we select the best depth in the least-squares sense.

Our efforts in deriving the first global reference model of Curie depth from magnetic anomalies give us a total number of 516,772 raw Curie depth estimates using the three different window sizes. Different window sizes emphasize different scales of Curie depth variations, but their average, as the final reference model, reduces random noises and improves the resolution model data is available in the supplementary Information. The final spatial resolution of the Curie depth model should be smaller than the smallest moving step, which is 49.4 km. The gridding resolution of our Curie depth model is 10 minutes.

## The Global Curie Depth Model (GCDM)

### Oceanic domain

Our global Curie depth model (GCDM) shows evident shallowing along mid-ocean ridges, particularly in the Indian, north Atlantic, and Arctic Oceans (Fig. 1). This reflects the active magmatism and upwelling of the hot asthenosphere. GCDM confirms the early results in the north Atlantic^11^, that in the background of increasing Curie depths with oceanic crustal ages^22^, anomalous off-ridge upwelling and oscillations in Curie isotherm are observed in all major sea basins, and in most cases, they are associated with small-scale convection or hotspots. For example, the Hawaii seamount trail, the Bermuda Rise, and the Cape Verde Islands all show small Curie depths.

**Figure 2:**
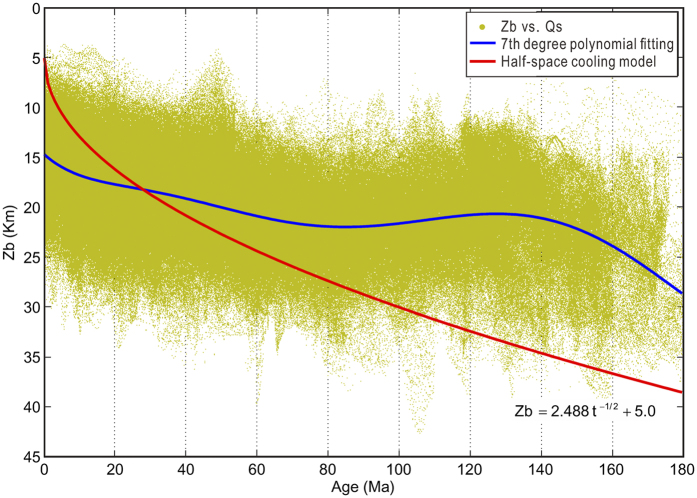
Curie depth (Zb) variation with oceanic crustal ages (t). The equation is based on the half-space cooling model assuming an average thermal diffusivity κ of 0.319 mm^2^/s or 10.06 km^2^/Ma^11^.

In both the Pacific and Atlantic, large-scale upwells in Curie points are observed in the age range from ~100 to 150 Ma (Fig. 1). By plotting all Curie depth estimates against oceanic crustal ages, we found that this large-scale Curie depth anomaly persists globally (Fig. 2). Assuming an average lithospheric thermal diffusivity κ of 0.319 mm^2^/s (or 10.06 km^2^/Ma), observed average Curie depths are larger than predicted from the half-space cooling model for ages <~30 Ma, but are smaller for older crustal ages. This implies that, near the mid-ocean ridges, temperatures are lower than that from the model prediction, probably due to stronger hydrothermal activities, but deep thermal perturbations exist in older lithospheres, particularly in the age range of ~100–150 Ma.

**Figure 3:**
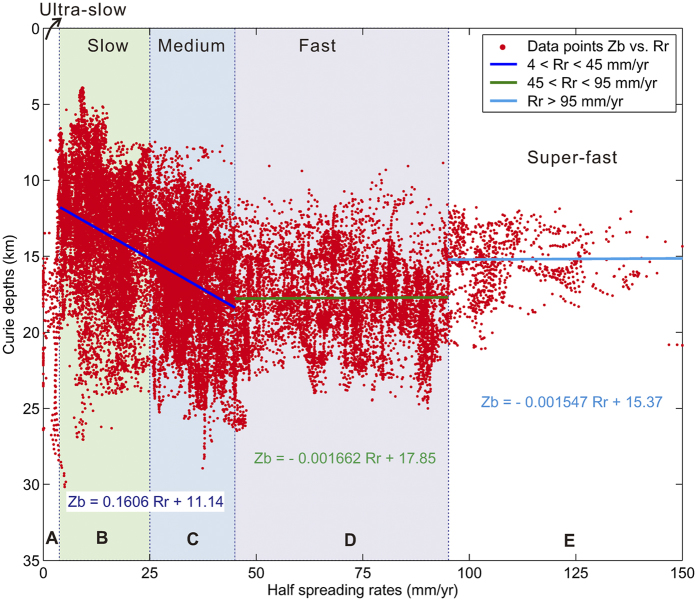
Curie depth (Zb) variation with spreading rates (Rr) within the 1 Ma isochrons of active spreading centers. We also find that the same pattern holds for data within the 5 Ma isochrones.

We find that Curie depths within the two 1 Ma isochrons of active ridges are strongly dependent on the spreading rate (Rr) of Müller *et al*.^22^. Ultra-slow spreading ridges (Rr < 4 mm/yr) show large Curie depths averaged at ~17.5 km (Fig. 3). However, the average Curie depth decreases abruptly at slightly higher rates, and then with increasing spreading rates from about 4 to 45 mm/yr (slow to medium spreading rate), the average Curie depth increases linearly, from ~12 to ~18 km. Along fast spreading ridges, average Curie depths decrease very slowly with rates and keep almost constant at ~17.7 km. Likewise along super-fast ridges, average Curie depths also decrease very slowly with spreading rates, but keep at a slightly smaller constant at ~15.2 km and with a narrower distribution. If the fractal scaling factors of magnetization are not dependent on the spreading rate and the Curie temperatures are constant, this overall complex dependence of Curie depths on the spreading rate indicate that the near-ridge thermal structures vary considerably with spreading rate.

The following hypothesis may be perceived to interpret these observations:

- For ultra-slow spreading ridge, the magma chamber may be deep seated or may not even exist, and slow mantle exhumation and sufficient cooling lead to large Curie depths.
- After the threshold of ~4 mm/yr, decompressive magmatism starts to prevail, and magma chamber form at shallow depth. With increasing spreading rate, decompressive partial melting could occur at progressively larger depths, and the crustal thickening from intensified magmatism can also result in deepening in Curie points. Increasing spreading rate may also trigger stronger hydrothermal circulation, which lowers down the temperature.
- Along both fast (45 < Rr < 95 mm/yr) and super-fast spreading centers (Rr > 95 mm/yr), their almost constant average Curie depths suggest that the spreading rate no longer exerts significant role on the thermal structure of the ridge within these respective rate ranges. But the apparent reduction in the average Curie depth around the spreading rate of 95 mm/yr indicates that thermal gradients increase again along super-fast ridges, to be similar to those at slow to medium spreading rate of 25 mm/yr. Super-fast ridges have the most active magmatism that can induce relatively shallower Curie depths.

### Continental domain

The majority of the largest Curie depths are found on the continents, most noticeably South America, Africa, and Indian Shield (Fig. 1). However, small Curie depths are also found within some old and stable cratons, such as the Northern China, Siberia, and North America, where large Curie depths are expected from their old ages. This may reflect that reactivation of these old continental cratons has induced thermal perturbations at depth. North China craton, for example, may have been reactivated by the subduction of the Pacific Plate^23^. The zone of small Curie depths along the western margin of America is also caused by active subductions and related arc magmatism and extension. In southern Africa, a large patch of shallow Curie points could be induced by hot spot magmatism, so is the noticeable Curie depth anomaly associated with the large intraplate hot spot Ahaggar Swell in northern Africa.

**Figure 4:**
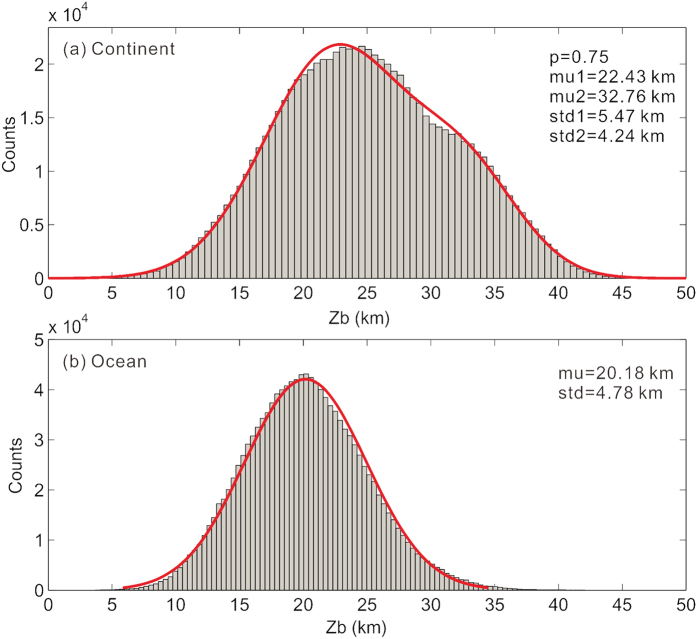
Distributions of Curie depths from continents (**a**) and oceans (**b**). Continental distribution can be fitted with a mixture of two normal distributions (red curve). Based on the maximum likelihood optimization, the mixing percentage (p) is 0.75 for the first normal distribution with a mean (mu1) of 22.43 km and a standard deviation (std1) of 5.47 km, and the second normal distribution has a mean (mu2) of 32.76 km and a standard deviation (std2) of 4.24 km. Oceanic distribution can be best fitted with a single normal distribution (red curve) with a mean (mu) of 20.18 km and a standard deviation (std) of 4.78 km.

Oceanic Curie depths show a good normal distribution with a smaller mean of 20.18 km and a smaller standard deviation than continental Curie depths (Fig. 4). By fitting continental Curie depth distribution with a mixture of two normal distributions, we find that the distribution is nearly bimodal (Fig. 4), which shows a first peak depth of 22.43 km, but a minor second peak occurs around 32.76 km, giving a tail of large Curie depths.

## Estimates of global heat loss

Previous estimates of global heat loss based on surface heat flow range widely from ~29 TW to 47 TW^1^,^2^,^3^,^4^,^5^,^6^. We further exam this problem with reference to our global Curie depth model.

### Global heat flow average

**Figure 5:**
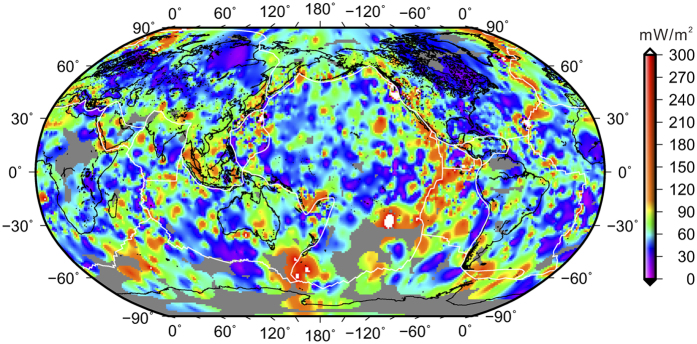
Our global heat flow model gridded using a 1° interval. White lines mark the major plate boundaries^32^. Map is generated using the software GMT version 5.2.1 (http://gmt.soest.hawaii.edu/)^31^.

**Figure 6:**
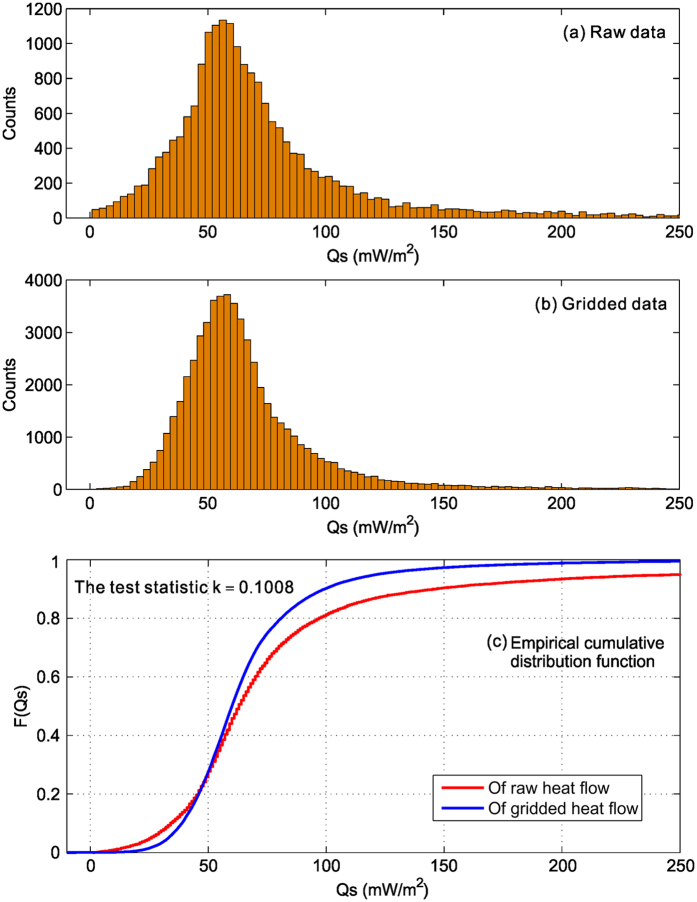
Distributions of global heat flow measurements before (**a**) and after gridding (**b**). We performed a Kolmogorov Smirnov statistical test to compare the two distributions (**c**). At the 5% significance level, the test rejected the null hypothesis that the raw and gridded heat flow are from the same continuous distribution.

Surface heat flow measures the heat loss at the surface of the earth; it could be modulated by hydrothermal circulation or sedimentary cover, but if evenly and densely distributed, its global average quantifies the global heat loss. Large-interval gridding also reduces local measurement biases in heat flow^11^,^20^, and hence increases the reliability of interpretation. Our heat flow model, based on a constant 1° interval gridding of all the raw data from the International Heat Flow Commission Database (http://www.heatflow.und.edu/; last updated in January 2011), captures the characteristics of main tectonic features (Fig. 5). Averaging of heat flow values is based on block averaging of (x, y, z) data by L2 norm. We estimate a mean position and heat flow value for every non-empty block in each 1° × 1° grid region to avoid aliasing short wavelengths. We then apply a constant 1° interval gridding of the averaged heat flow using the minimum curvature algorithm with a tension factor of 0.5. Histograms show that gridded heat flow reduces the number of extremely low and high heat flow values caused by hydrothermal process, and retains the original statistical mean (Fig. 6a,b). At the 5% significance level, the Kolmogorov Smirnov statistical test rejected the null hypothesis that the raw and gridded heat flow are from the same continuous distribution (Fig. 6c).

If we interpolate all the raw heat flow data in a constant 100 km interval, using the minimum curvature algorithm with a tension factor of 0.5, the global heat flow average is 67.8 mW/m^2^. Alternatively, we interpolate the same data set in a constant 1° interval (Fig. 5), and then resample the grid in a constant 10 minute interval. Then in each 1° × 1° quadrangle we estimate the average of the gridded heat flow, and the global mean from these quadrangles is 68.3 mW/m^2^. These values are substantially smaller than some of the recent estimates (e.g., 91.6 mW/m^2^)^24^.

### Global correlation between heat flow and Curie depth

Previous regional studies have shown that heat flow and Curie depth are correlated, following a theoretical thermal conduction relationship^10^,^11^. By comparing the Curie depth map (Fig. 1) with heat flow map (Fig. 5), we observe that this correlation should hold for the global data, particularly in active tectonic areas such as mid-ocean ridges and subduction zones. In old and stable continental cratons, surface heat flow may deviate from deep thermal structures due to their long evolutional histories and late-stage thermal perturbations. In thick continental lithosphere, there may be long delays between changes in asthenosphere temperature and measurable effects arriving at the surface^25^,^26^. Shallow radioactive contribution to surface heat flow is also larger in the continental domains.

**Figure 7:**
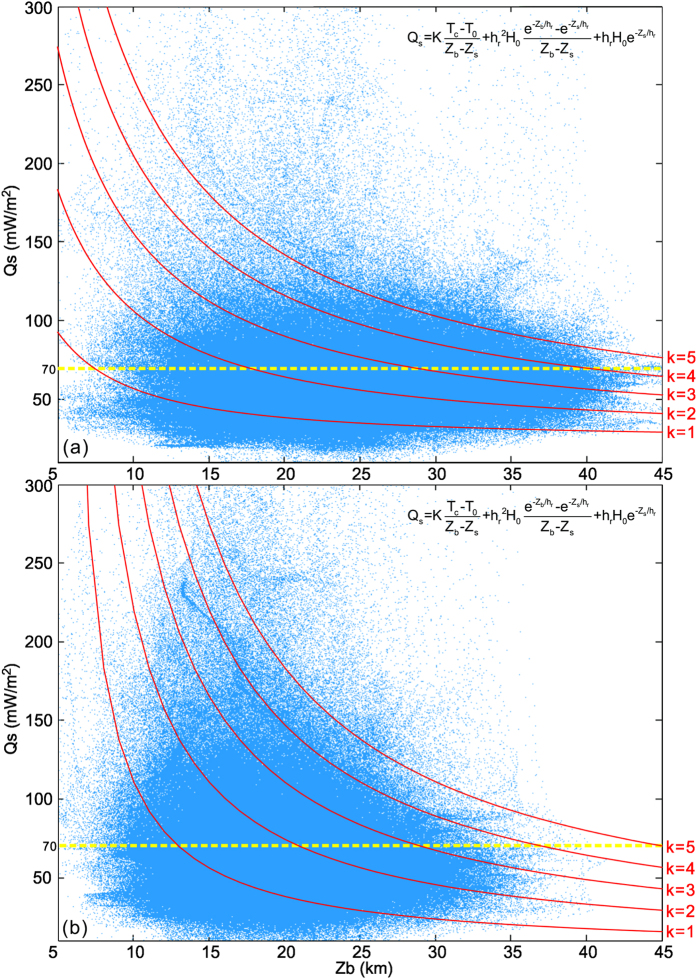
Global correlation between heat flow and Curie depths for (**a**) continental domain and (**b**) oceanic domain, respectively. In the equation, Q_s_ is the surface heat flow, T_c_ is the Curie temperature at the Curie depth Z_b_, T_0_ is the temperature at the surface elevation Z_s_, K is the average thermal conductivity of the magnetic layer, H_0_ is the heat production rate at the surface, and h_r_ is the characteristic drop-off of heat production. For oceanic lithosphere, we assume H_0_ = 1.37 μW/m^3^, h_r_ = 5.0 km, T_c_ = 550 °C, T_0_ = 5 °C, and Z_s_ = 4 km. For continents, we take H_0_ = 2.0 μW/m^3^, hr = 10.0 km, and Zs = −1 km to account for the larger radioactive contribution^23^. The yellow dashed line marks the global heat flow average estimated in this paper.

Figure 7 Shows a large scattering in the global correlation between heat flow and Curie depth; this is expected since there are diverse geological units and local structures. Nevertheless, good correlations between heat flow and Curie depth can be observed, i.e., high heat flow measurements tend to correlate with small Curie depths, and vice versa. It is seen that most continental points are clustered in the vicinity of the continental theoretical curve with an average thermal conductivity K of ~2.5 W(m°C)^−1^, and most oceanic data can be best fitted with the oceanic theoretical curve of an average K = ~2.0 W(m°C)^−1^. These conductivities are compatible with those of granite and basalt^27^,^28^.

### Global heat loss

The Earth is divided into 360 × 180 bins of 1° × 1° in size. The exact surface area (A_i_) of these latitude-longitude quadrangle bins are calculated assuming a spherical ellipsoid. Calculated heat flow data from Curie depths are gridded in a 10 minute interval. The surface heat flow measurements are interpolated using a constant 1° interval and the grid is also resampled in a 10 minute interval. These data are then averaged, respectively, within each 1° × 1° latitude-longitude quadrangle (Q_si_). Quadrangles without heat flow measurements or Curie depth constraints are assumed to take the global average of heat flow. The total heat loss (T_hl_) is then calculated by


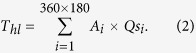


We find that the measured global heat loss is 3.46 × 10^13^ W (or 34.6 TW) based on our interpolated heat flow. Calculated conductive heat flow from Curie depths using an average continental thermal conductivity k = 2.5 W(m°C)^−1^ and an oceanic average k = 2.0 W(m°C)^−1^, is averaged at 72.125 mW/m^2^. Here the radioactive heat contribution is included in the estimation. The global heat loss hereby obtained is 3.66 × 10^13^ W (or 36.6 TW). These values are much lower than some of the early estimates^3^, but slightly larger than some others^2^,^5^,^6^.

## Conclusions

Our global Curie depth model (GCDM) shows that oceanic Curie depths increase with crustal ages, but deviate from the half-space cooling model in both young and old oceanic lithospheres. Measured Curie depths are larger than the model predictions in young oceanic lithosphere of age <~30 Ma, but are smaller in older lithospheres. In particular, there is a significant decrease of Curie depths for crustal ages between ~100 and 150 Ma, noticeable in both Atlantic and Pacific. These deviations reflect strong hydrothermal circulations in young oceanic lithospheres and thermal perturbations in aged oceanic lithospheres.

Along active mid-ocean ridges, our study shows that Curie depths are strongly dependent on the spreading rate. There is a unique Curie depth distribution for each single spreading-rate type (i.e., ultra-slow, slow, medium, fast, and super-fast ridges), indicating strong coupling between spreading rate and seafloor-spreading mechanisms, thermal structures, and hydrothermal activities.

Continental domains have the largest Curie depths, but also show nearly a bimodal distribution; small Curie depths are found in some of old continental cratons, indicating that deep thermal reactivations caused inconsistencies between Curie depth and surface heat flow.

Despite a large scatter of points, global correlations between Curie depth and gridded surface heat flow in 1° interval follow theoretical curves of an average thermal conductivity of K = 2.0 W(m°C)^−1^ for the oceanic data and an continental average K = 2.5 W(m°C)^−1^. With these optimal conductivities, the calculated global heat flow average is 72.125 mW/m^2^. This estimate is close to measured global heat flow average from direct large-interval gridding of raw heat flow data, which is about 68.0 mW/m^2^. We propose that the global heat flow average is around 70.0 mW/m^2^ and the global heat loss ranges from ~34.6 to 36.6 TW.

## Additional Information

**How to cite this article:** Li, C.-F. *et al*. A global reference model of Curie-point depths based on EMAG2. *Sci. Rep.* **7**, 45129; doi: 10.1038/srep45129 (2017).

**Publisher's note:** Springer Nature remains neutral with regard to jurisdictional claims in published maps and institutional affiliations.

## Supplementary Material

Supplementary Dataset 1
